# DECODING CENTENARIAN DAILY ACTIVITIES: A REAL-LIFE SENSOR APPROACH

**DOI:** 10.1093/geroni/igae098.0217

**Published:** 2024-12-31

**Authors:** Melanie Stahlmann, Tabea Buehrer, Christina Roecke, Daniela Jopp, Stefano Cavalli, Armin von Gunten, Francois Herrmann, Mike Martin

**Affiliations:** University of Zurich, Zurich, Zurich, Switzerland; University of Zurich, Zurich, Zurich, Switzerland; University of Zurich, Zurich, Zurich, Switzerland; University of Lausanne, Lausanne, Vaud, Switzerland; University of Applied Sciences and Arts of Southern Switzerland, Manno, Ticino, Switzerland; Lausanne University Hospital, Prilly, Vaud, Switzerland; Hopitaux Universitaires Geneve, Thonex, Geneve, Switzerland; University of Zurich, Zurich, Zurich, Switzerland

## Abstract

In response to the decade of healthy aging, understanding daily activities in older adults has become increasingly important in aging research. While ambulatory assessment is now quite common, it often misses the oldest age groups. The SWISS100 study is the first to fill this gap by employing real-life sensors to explore Swiss centenarians’ lives, blending questionnaire data with actigraphs for physical activity assessment, and smartphones equipped with the Electronically Activated Recorder (EAR) to capture everyday sounds and conversations. Out of N = 44 German-speaking centenarians who consented to the EAR study, technical issues and other challenges led to a final dataset from 35 participants. Over a time period of four days, more than 10,000 audio clips were collected, equating to roughly 2.7 hours of recordings per participant, which is about 4% of their waking hours. These recordings were then transcribed and analyzed to identify activities and social interactions. The analyses revealed that centenarians primarily spend their time alone (85%), with TV watching and resting as their main activities. When interacting, centenarians mostly engage with one familiar person, with a significant portion of conversations being “substantive talk” (44%), followed by “practical talk” (34%), though notable variations exist among individuals. These findings offer a deeper understanding of Centenarians’ social and leisure patterns, enriching our knowledge of their daily lives. This study also demonstrates the feasibility and value of mobile sensing in researching very advanced ages, encouraging future studies to integrate such innovative methods for a more comprehensive view of participants’ real-life experiences.

